# Efficacy and Safety of a Revised Gastric Peroral Endoscopic Myo‐Neurotomy Procedure for Bariatric Endoscopy in a Porcine Model

**DOI:** 10.1002/deo2.70230

**Published:** 2026-01-09

**Authors:** Yasushi Yamasaki, Kenta Hamada, Junki Toyosawa, Akinobu Takaki, Takehiro Tanaka, Hiroyuki Okada, Motoyuki Otsuka

**Affiliations:** ^1^ Department of Gastroenterology Okayama University Hospital Okayama Japan; ^2^ Department of Pathology Okayama University Graduate School of Medicine Dentistry and Pharmaceutical Sciences Okayama Japan; ^3^ Department of Internal Medicine Japanese Red Cross Society Himeji Hospital Himeji City Japan

**Keywords:** bariatric endoscopy, body weight, intervention, obesity, peroral endoscopic myo‐neurotomy

## Abstract

**Background:**

Endoscopic sleeve gastroplasty is a minimally invasive and effective treatment for obesity, although gastric mucosa folding and subsequent reduced visibility of the gastric lining raise concerns for some patients. This study evaluates the safety and efficacy of a revised peroral endoscopic myo‐neurotomy (R‐POEM‐N) technique using a submucosal approach as an alternative bariatric procedure in a porcine model.

**Methods:**

Twelve pigs underwent either an R‐POEM‐N procedure (*n* = 8) or a sham endoscopic procedure (*n* = 4). Myo‐neurotomy was performed at four sites in the R‐POEM‐N group: two on the greater curvature of the gastric body and two in the antrum. All pigs were fed diets at twice the standard weight‐equivalent dose and observed for 8 weeks (8 W) following the procedure. The primary outcome was the proportional change in body weight at 8 W, calculated as the ratio of weight change to baseline weight. Secondary outcomes included changes in serum glucolipid levels, food intake, and the safety and duration of R‐POEM‐N.

**Results:**

R‐POEM‐N was successfully completed in all pigs without significant adverse events. The procedure time was 171.3 ± 3.0 min. The proportional change in body weight was significantly lower in the R‐POEM‐N group compared with the control group (*p* = 0.017). Food intake in the R‐POEM‐N group was significantly lower than that in the control group (*p* < 0.05). Changes in serum glycolipid levels were not significantly different between groups.

**Conclusions:**

R‐POEM‐N successfully reduced weight gain in pigs, supporting its potential as a novel bariatric intervention with minimal invasiveness.

**Abbreviations**
BEAM: bariatric endoscopic antral myotomy
GLP‐1: glucagon‐like peptide‐1
POEM: peroral endoscopic myotomy
POEM‐N: peroral endoscopic myo‐neurotomy
R‐POEM‐N: revised peroral endoscopic myo‐neurotomy.

## 1 Introduction

Obesity is strongly associated with diabetes mellitus, dyslipidemia, cardiovascular disease, and certain cancers [1], making it a rapidly growing global health and economic challenge [2]. As of 2022, approximately 878 million adults worldwide were affected by obesity [3].

Bariatric and metabolic endoscopy and surgery are proven interventions that achieve significant weight loss and improve metabolic conditions [4, 5, 6, 7]. Endoscopic sleeve gastroplasty has gained particular prominence due to its minimally invasive nature and proven efficacy [8, 9]. However, concerns have been raised about its potential to limit visibility of the gastric lining, as large areas of the mucosa are folded during the procedure [10]. For patients with high‐risk conditions for gastric cancer, such as atrophic gastritis or intestinal metaplasia, endoscopic sleeve gastroplasty may be inappropriate due to the increased risk of missing or misdiagnosing gastric cancer [11, 12]. Recently, the submucosal myotomy approach, which aims to suppress gastric peristalsis and delay gastric emptying, has garnered attention as a promising technique in bariatric and metabolic endoscopy [13, 14]. These procedures are adapted from the peroral endoscopic myotomy (POEM) technique that was originally developed for the treatment of esophageal achalasia [15]. To date, two such techniques have been described: bariatric endoscopic antral myotomy (BEAM), involving antral myotomy, and POEM‐N, involving antral myo‐neurotomy [13, 14].

In a previous animal study, we demonstrated the feasibility and safety of the POEM‐N procedure for bariatric and metabolic endoscopy [14]. However, several challenges remain, including the prolonged procedure time and suboptimal weight‐loss outcomes. To address these issues, we developed a revised POEM‐N (R‐POEM‐N) procedure, which incorporates elongated submucosal tunnels at the gastric body and antrum. This study evaluates the safety and efficacy of the R‐POEM‐N technique in an experimental porcine model.

## 2 Materials and Methods

### 2.1 Study Approval

This study was conducted in compliance with the Declaration of Helsinki and Japanese animal protection laws and was approved by the Animal Care and Use Committee of Okayama University (institutional number: OKU‐2021558).

### 2.2 Study Design

Twelve live, three‐way crossbred, 3‐month‐old female pigs were included in this study. On the day of the procedure, pigs were randomly assigned to either the R‐POEM‐N group (*n* = 8) or the control group (*n* = 4). The pigs underwent either the R‐POEM‐N or a sham endoscopic procedure. Following the procedure, all pigs were fed with the miniature swine diet MP‐A (ORIENTAL YEAST CO, LTD., Japan) at twice the amount of the weight‐equivalent standard dose (0–2 weeks: 1340 g/day; 3–5 weeks: 1670 g/day; 6–8 weeks: 1970 g/day). Body weight and serum glycolipid levels were measured at three time points: on the day of the procedure (0 W), 4 weeks post‐procedure (4 W), and 8 weeks post‐procedure (8 W). Liver lipid content was assessed through liver biopsy at 8 W prior to euthanasia.

### 2.3 Outcomes

The primary outcome was the proportional change in body weight, calculated as the ratio of weight change at 8 W to the baseline weight at 0 W. Secondary outcomes included changes in serum glucolipid levels, amount of food intake, the safety of the R‐POEM‐N procedure, and its duration. Safety was defined as successful completion of R‐POEM‐N without severe adverse events, such as significant changes in vital signs, marked appetite loss (as assessed by veterinarians), or peritonitis. Procedure time was recorded from the start of submucosal injection to the completion of clip closure.

### 2.4 Interventions

Following a 7‐day environmental acclimation period, pigs were fasted for 24 h prior to the procedure. Anesthesia was induced with an intramuscular injection of medetomidine (0.04 mg/kg), midazolam (0.2 mg/kg), and ketamine (5 mg/kg), followed by tracheal intubation and maintenance with 2%–5% inhaled isoflurane.

The R‐POEM‐N procedure, as detailed below, was performed at four sites: two on the greater curvature of the gastric body and two in the antrum. The sham procedure performed in the control group consisted of endoscopic gastric observation under anesthesia without creating a submucosal tunnel. Post‐procedure, all pigs were allowed water and resumed the MP‐A diet the following day. No antibiotics were administered during the study period. Veterinarians closely monitored eating habits, body weight, and overall condition throughout the study period. At 4 and 8 W, all pigs underwent endoscopy under general anesthesia to evaluate the procedure site. At 8 W, following the withdrawal of the endoscope, a midline celiotomy was surgically performed to visually assess the presence or absence of peritonitis and to assess the peritoneal aspect of the R‐POEM‐N site. The right lobe of the liver was collected from all pigs prior to euthanasia by exsanguination.

### 2.5 Revised POEM‐N

**FIGURE 1:**
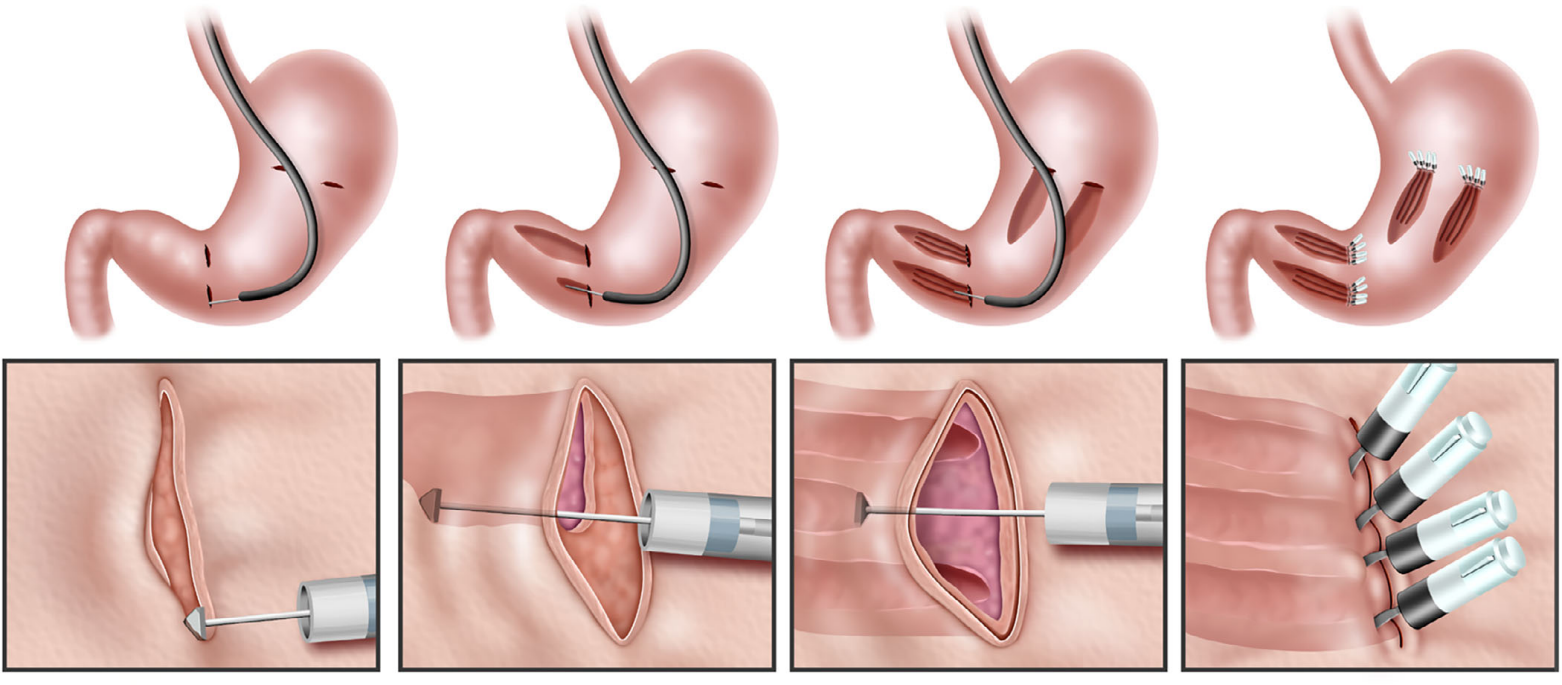
Schema of the revised peroral endoscopic myo‐neurotomy procedure. First, submucosal incision. Second, creation of a submucosal pocket. Third, myo‐neurotomy. Lastly, entry closure with clips.

**FIGURE 2:**
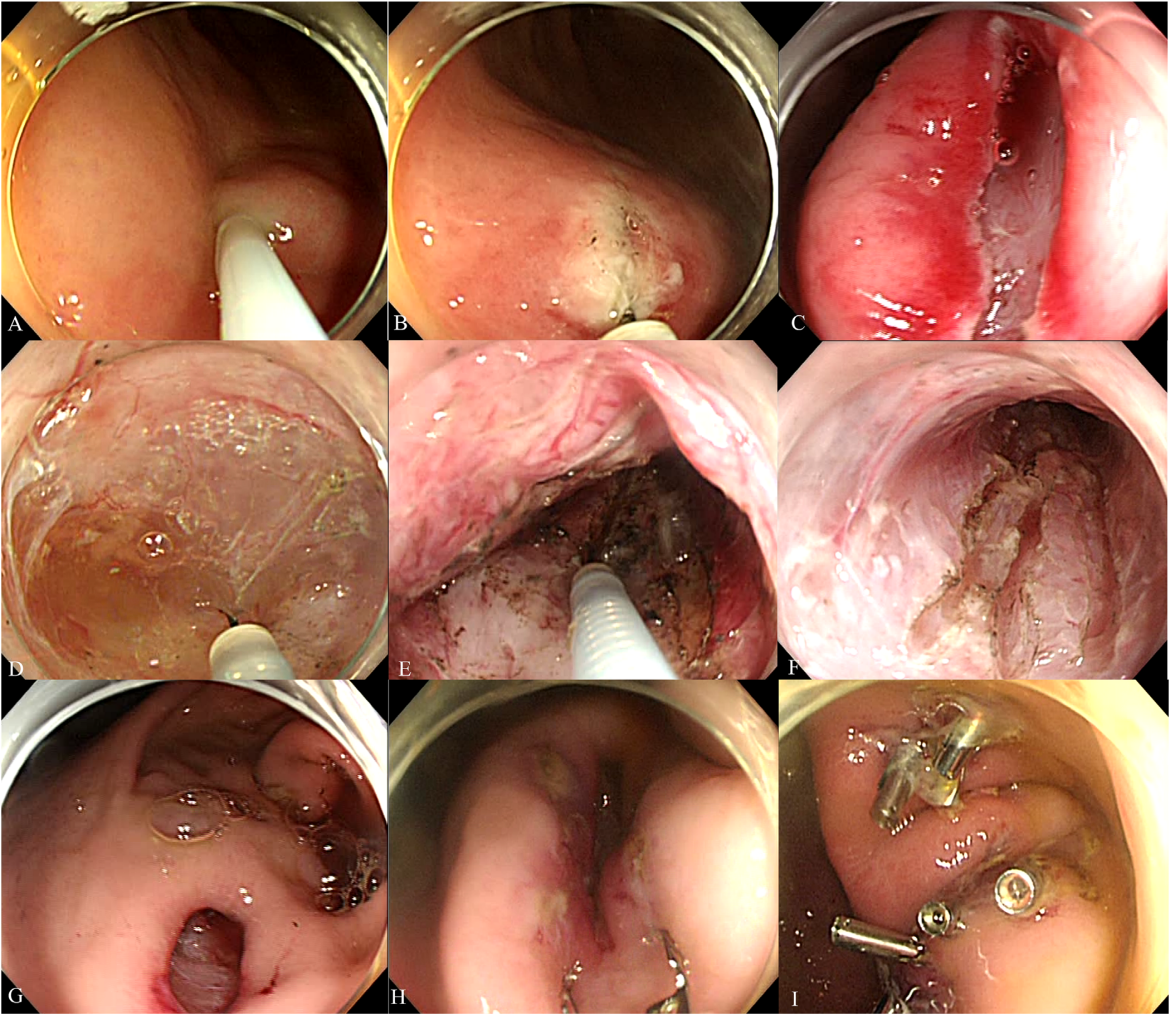
Intra‐operative images of the revised peroral endoscopic myo‐neurotomy procedure. (A) Submucosal injection. (B, C) Longitudinal incision. (D) Creating a submucosal pocket. (E, F) Myo‐neurotomy. (G–I) Entry closure.

Figures 1 and 2, along with Video S1, illustrate the POEM‐N procedure. R‐POEM‐N was performed initially at the greater curvature of the antrum, followed by the greater curvature of the gastric body. A single‐channel endoscope (PCF‐Q240ZI; Olympus, Tokyo, Japan), equipped with a disposable attachment and utilizing CO_2_ insufflation, was advanced into the stomach. Following irrigation of the stomach with physiological saline, a submucosal injection of saline was performed at the greater curvature of the antrum. A 2 cm longitudinal mucosal incision was made, and submucosal dissection was performed to create entry into the submucosal tunnel using a triangle‐tip electrosurgical knife (TT knife; Olympus). Mucosal incision and submucosal dissection were performed using the VIO300D (ERBE; Tübingen, Germany) in high‐cut mode (effect 3, 30 W) and spray mode (effect 2, 50 W), respectively. The endoscope was advanced into the tunnel, and submucosal dissection was performed just above the muscle layer, extending distally to create a 5‐cm submucosal tunnel with preservation of the mucosal layer. The distal extent of the dissection was stopped 2 cm proximal to the pyloric ring. The extension of the submucosal tunnel was adjusted based on the known length of the TT knife, allowing a consistent 5‐cm dissection. Once the muscle layer within the submucosal tunnel was exposed, the circular muscle was incised longitudinally using spray mode (effect 2, 50 W) to perform the myo‐neurotomy. Auerbach's plexus, located just beneath the circular muscle, was targeted and ablated using the spray mode. The longitudinal myo‐neurotomy was performed three times within each tunnel. When precise control of the cutting depth was challenging, the depth of the myo‐neurotomy was adjusted by gently hooking and cutting the circular muscle fibers with the tip of the TT knife, ensuring accurate exposure and ablation of Auerbach's plexus. Finally, the endoscope was withdrawn from the tunnel, and the entry site was securely closed with endoscopic clips. This procedure was repeated at all four sites.

If intraprocedural perforation occurred during myo‐neurotomy, leading to abdominal compartment syndrome due to elevated abdominal pressure, an abdominal puncture was performed using a 20‐gauge injector needle to release gas from the abdominal cavity and reduce pressure.

### 2.6 Statistical Assessment

Statistical analysis was performed using JMP 17 software (SAS Institute, Cary, NC, USA). Continuous variables are presented as mean (± standard deviation). Mann‐Whitney's U test was used to compare continuous data, and a *p*‐value < 0.05 was considered statistically significant.

## 3 Results

### 3.1 Safety and Duration of R‐POEM‐N Procedure

**FIGURE 3:**
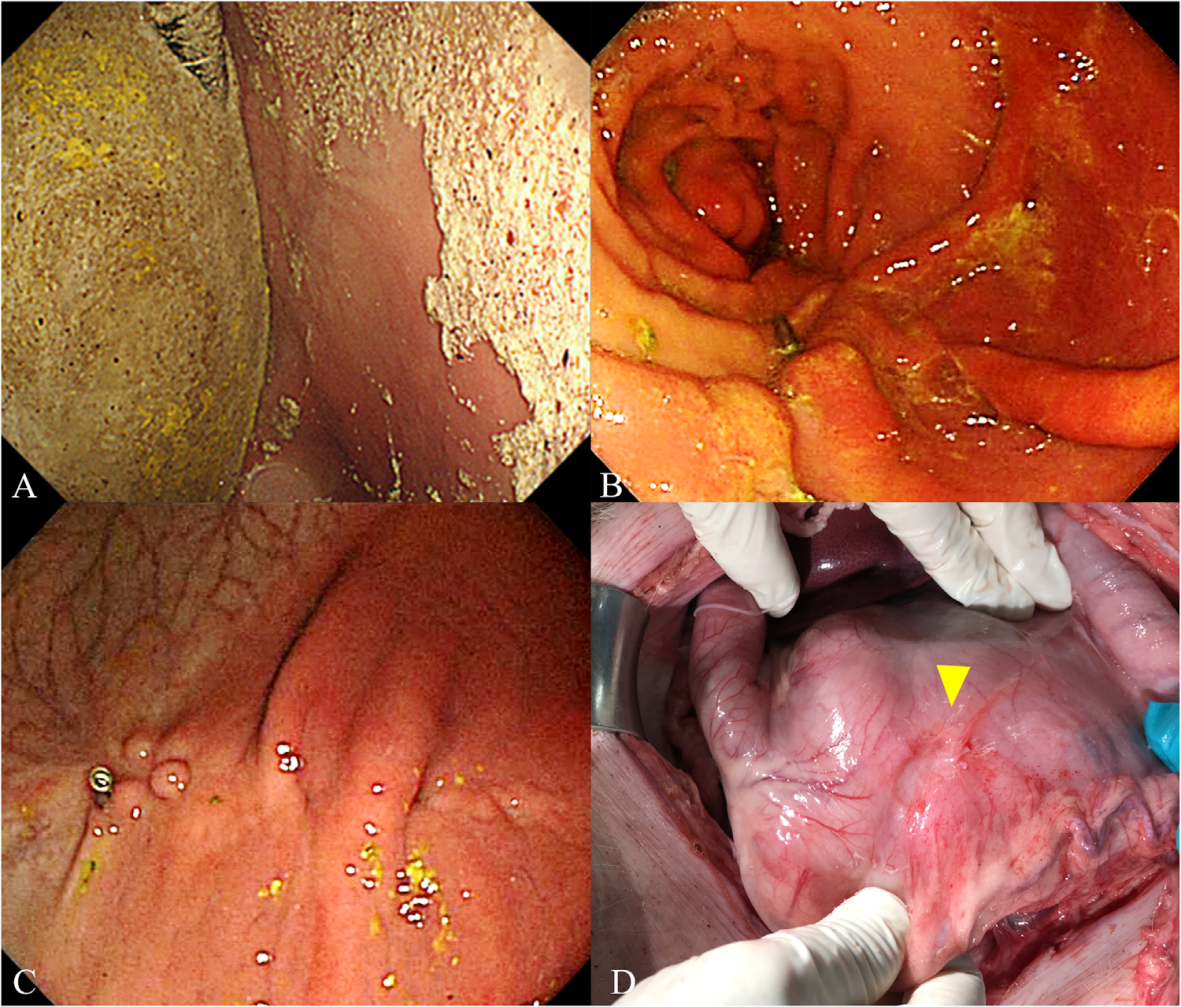
Endoscopic and laparotomic images at 8 weeks post‐procedure. (A) Food residue in the stomach. (B) Antral incision site scar. (C) Gastric body incision site scar. (D) Mild adhesions on the peritoneal side of the stomach during euthanasia.

The outcomes of the R‐POEM‐N procedure are detailed in Table 1. All R‐POEM‐N procedures were successfully completed without significant adverse events, and the clinical course progressed favorably. The mean procedure time for R‐POEM‐N was 171.3 (± 3.0) min. Abdominal punctures were required in four pigs (50%) during the R‐POEM‐N procedure. At 8 W, endoscopy revealed large amounts of food residue in the stomachs of R‐POEM‐N pigs (Figure 3A), and the incision sites from R‐POEM‐N were visualized and appeared well‐healed (Figure 3B,C). Mild adhesions were observed on the peritoneal side of the stomach in two R‐POEM‐N pigs (25%) at 8 W prior to euthanasia (Figure 3D), but no signs of peritonitis were detected.

**TABLE 1:** Outcomes of the revised peroral endoscopic myo‐neurotomy procedure.

	R‐POEM‐N (*n* = 8)
Mean procedure time, minutes	171.3
Total clips used to close entry sites, *n*	19
Intra‐operative perforation requiring abdominal puncture, *n*	4 (50%)
Intraperitoneal adhesions, *n*	2 (25%)
Favorable clinical progression, *n*	8 (100%)

*Note*: R‐POEM‐N; revised peroral endoscopic myo‐neurotomy.

### 3.2 Changes in Body Weight and Glycolipid Levels

**FIGURE 4:**
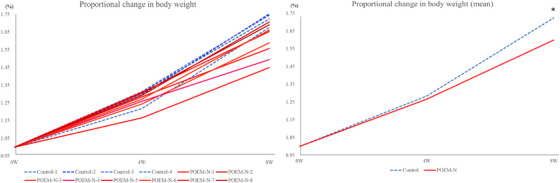
Proportional change in body weight. (A) Change in each pig. (B) Mean proportional change in each group (*p* = 0.017).

Figures 4A and 4B show the proportional changes in body weight in the control and R‐POEM‐N groups. The mean proportional change in body weight was significantly slower in the R‐POEM‐N group compared with the control group (*p* = 0.017).

Table 2 displays the absolute levels of all evaluated parameters at 0, 4, and 8 W. Changes in serum cholesterol, triglyceride, glucose, and Glucagon‐like peptide‐1 (GLP‐1) levels were not significantly different between the control and R‐POEM‐N groups. Similarly, liver cholesterol and triglyceride levels showed no significant differences between the groups.

**TABLE 2:** Absolute levels of evaluated parameters.

	Control (*n* = 4) mean ± SD	R‐POEM‐N (*n* = 8) mean ± SD	*p*‐Value
Body weight, kg, 0 W	27.5 ± 1.0	29.8 ± 1.7	
Body weight, kg, 4 W	35.4 ± 2.4	37.8 ± 1.9	
Body weight, kg, 8 W	47.5 ± 2.6	47.7 ± 2.8	
Weight change rate, %, 8 W‐0 W/0 W	72.5 ± 3.5	60.1 ± 9.4	0.017
Serum cholesterol, mg/dL, 0 W	122.8 ± 34.0	101.5 ± 18.2	
Serum cholesterol, mg/dL, 4 W	94.8 ± 5.9	99.9 ± 9.7	
Serum cholesterol, mg/dL, 8 W	88.3 ± 4.6	93.9 ± 9.3	
Cholesterol change, mg/dL, 8 W‐0 W	−34.5 ± 30.8	−7.6 ± 18.4	0.074
Serum triglyceride, mg/dL, 0 W	31.8 ± 8.4	23.6 ± 11.8	
Serum triglyceride, mg/dL, 4 W	20.0 ± 9.5	13.5 ± 9.7	
Serum triglyceride, mg/dL, 8 W	16.3 ± 9.5	6.3 ± 3.4	
Triglyceride change, mg/dL, 8 W‐0 W	−15.5 ± 12.7	−17.4 ± 12.6	0.609
Serum glucose, mg/dL, 0 W	120.0 ± 29.1	91.5 ± 16.6	
Serum glucose, mg/dL, 4 W	84.0 ± 14.1	93.0 ± 10.4	
Serum glucose, mg/dL, 8 W	82.3 ± 12.5	101.3 ± 7.9	
Glucose change, mg/dL, 8 W‐0 W	−1.75 ± 9.9	8.3 ± 8.7	0.088
Serum GLP‐1, pmol/L, 0 W	1.5 ± 0.4	0.8 ± 0.3	
Serum GLP‐1, pmol/L, 8 W	1.1 ± 0.4	0.7 ± 0.5	
GLP‐1 change, pmol/L, 8 W‐0 W	−0.3 ± 0.0	−0.06 ± 0.5	0.305
Liver cholesterol, mg/g, 8 W	3.2 ± 0.3	3.2 ± 0.3	1.000
Liver triglyceride, mg/g, 8 W	10.6 ± 3.6	8.2 ± 2.8	0.232

*Note*: GLP‐1, Glucagon‐like peptide‐1; R‐POEM‐N, revised peroral endoscopic myo‐neurotomy; SD, standard deviation.

### 3.3 Food intake

**FIGURE 5:**
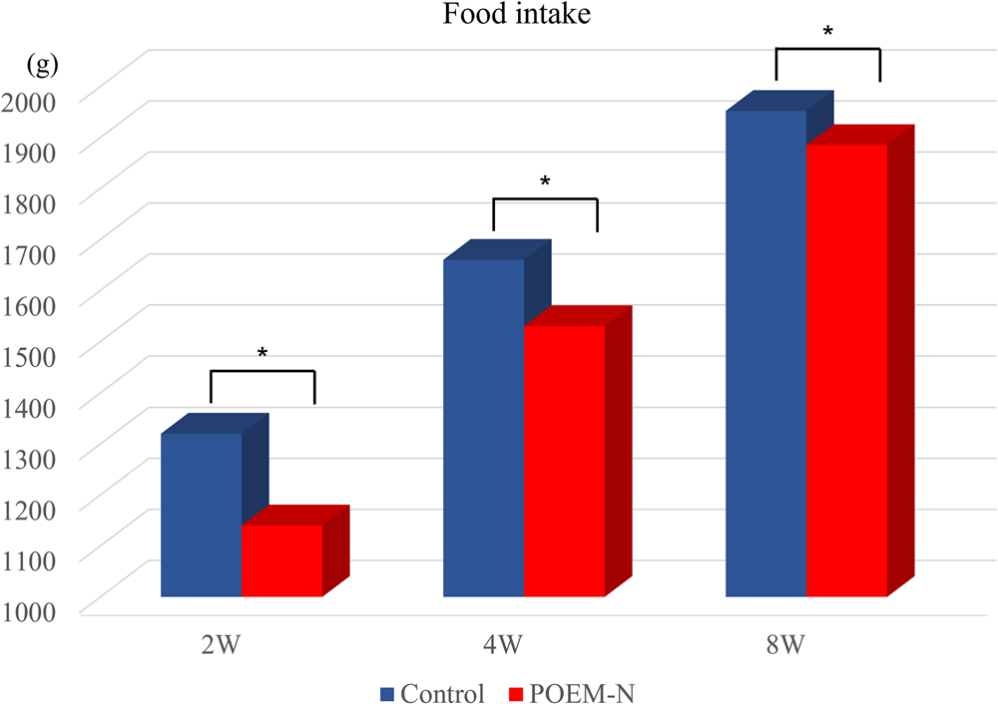
Amount of food intake. Significant differences in food intake were noted between groups at each time point (2 W: *p* = 0.006; 4 W: *p* = 0.010; 8 W: *p* = 0.028).

Figure 5 illustrates the mean food intake in both groups at 0, 4, and 8 W. The mean food intake in the R‐POEM‐N group was significantly lower than that in the control group at each time point.

## 4 Discussion

This study illustrates the safety and efficacy of the R‐POEM‐N procedure in a porcine model. R‐POEM‐N significantly suppressed weight gain in growing pigs without causing severe adverse events.

R‐POEM‐N decreased the slope of weight gain in this study over the eight‐week observation period, which is primarily attributed to a reduction in food intake. Theoretically, myo‐neurotomy suppresses gastric peristalsis, leading to decreased gastric emptying, abdominal distension, and reduced appetite. A previous report on an antral myotomy technique demonstrated a 68% delay in gastric emptying time compared with baseline, as measured by a gastric emptying breath test [13]. Additionally, our initial report on the POEM‐N technique demonstrated histopathologic damage to Auerbach's plexus, thereby reducing gastric peristaltic contractions [14]. Although the present study did not evaluate histopathological alterations in Auerbach's plexus or changes in peristaltic activity after R‐POEM‐N, these previous findings support the rationale that R‐POEM‐N may reduce gastric peristalsis. However, whether the localized damage to Auerbach's plexus induced by R‐POEM‐N truly contributes to sustained suppression of gastric peristalsis remains uncertain and should be further investigated in future studies.

From a technical standpoint, R‐POEM‐N is more feasible because it employs a narrow submucosal tunnel, which is technically easier to perform and close than the wide tunnel used in the previously reported antral myotomy [13]. Moreover, R‐POEM‐N is modular—additional submucosal tunnels can be created at other gastric sites if necessary. Furthermore, scar formation following myo‐neurotomy may result in localized gastric deformity, which could further contribute to a reduction in gastric volume.

Regarding the safety and tolerance of the procedure, all pigs remained in good health following R‐POEM‐N. Although abdominal puncture was required in 50% of the pigs due to increased intra‐abdominal pressure caused by perforation during myo‐neurotomy, none of the pigs experienced worsening vital signs. During R‐POEM‐N, careful attention was given to avoid damaging the mucosal flap, following the same principles as the original POEM procedure [15]. Intra‐operative perforation did not result in any serious delayed adverse events, as the mucosal flap was not exposed to thermal injury and the entry site was completely closed with clips. Mild adhesions were observed in 25% of the pigs but were easily released manually during peritoneal examination at 8 W.

The procedure time for R‐POEM‐N was shorter compared with that of conventional POEM‐N, which typically exceeded 3 h [14]. The conventional method required a lateral submucosal incision to access the muscle layer to create the submucosal pocket, a technically demanding and time‐consuming approach. In contrast, the revised method accessed the muscle layer by creating a longitudinal submucosal tunnel, which is technically simpler and requires less time. R‐POEM‐N included myo‐neurotomy at four sites, which was twice as many as conventional POEM‐N; however, the procedure time for R‐POEM‐N remained acceptable. However, compared with endoscopic sleeve gastroplasty, which typically requires only about 1 h [16], further improvements are necessary to reduce the procedure time of R‐POEM‐N.

While this study demonstrates the efficacy of R‐POEM‐N in an animal model, it has several limitations. First, growing pigs were used as the experimental model, which does not fully capture the potential weight‐loss effects of R‐POEM‐N. Although an obese diabetic pig model fed a high‐fat diet during the study period would theoretically be more suitable, conducting comparative experiments using such a model is challenging because of its high cost. Second, this was a pilot study; therefore, the sample size of the experimental models was not calculated, and the follow‐up period was limited. A larger sample size with a longer follow‐up period will be necessary to more accurately evaluate the long‐term effects of R‐POEM‐N. In addition, a comparative study between R‐POEM‐N and a control group undergoing submucosal tunneling without myo‐neurotomy will also be needed. This step will be essential for exploring the potential application of R‐POEM‐N as a bariatric intervention in humans. Third, the optimal sites for myo‐neurotomy remain unclear based on this study. Because creating a long submucosal tunnel along the greater curvature is theoretically challenging, the procedure was performed at four sites on the greater curvature in this study. However, the efficacy of performing a long submucosal tunnel at only two sites on the greater curvature of the antrum should be further evaluated, as the antrum plays a central role in gastric peristalsis and emptying. Fourth, changes in serum glycolipid levels could not able to be evaluated because, despite receiving twice the standard feed amount, control pigs did not exhibit glycolipid accumulation. To adequately evaluate changes in serum glycolipid levels in future studies, a higher dietary intake may be required. Previous human studies of other bariatric and metabolic endoscopic procedures have demonstrated improvements in glycolipid metabolism alongside reductions in body weight [17, 18].

In conclusion, R‐POEM‐N successfully reduced weight gain in a porcine model, warranting further investigation as a potential bariatric and metabolic treatment in humans.

## Author Contributions

Yasushi Yamasaki, Kenta Hamada, and Akinobu Takaki contributed to the study conception and design. Takehiro Tanaka performed the pathological diagnosis. Yasushi Yamasaki, Kenta Hamada, and Junki Toyosawa performed the procedure. Yasushi Yamasaki wrote the first draft of the manuscript, and Hiroyuki Okada and Motoyuki Otsuka revised the manuscript. All the authors read and approved the final version of the manuscript.

## Ethics Statement

**Approval of the research protocol by an Institutional Reviewer Board**: The study protocol was approved by the Animal Care and Use Committee, Okayama University (institutional number: OKU‐2021558).

## Consent

N/A.

## Conflicts of Interest

The authors declare no conflicts of interest.

## Funding

This work was supported by Grants‐in‐Aid for Scientific Research (JSPS KAKENHI Grant Number JP21K15999 to Yasushi Yamasaki).

## Clinical Trial Registration

N/A.

## Animal Studies

This study was conducted in compliance with the Declaration of Helsinki and Japanese animal protection laws.

## Supporting information




**Video S1**: Procedure of revised peroral endoscopic myo‐neurotomy (R‐POEM‐N).

## Data Availability

All data generated or analyzed during this study are included in this article. Further inquiries can be directed to the corresponding author.
